# Functionality of *Toxoplasma gondii* antibodies in a population of Beninese pregnant women exposed to malaria

**DOI:** 10.1038/s41598-025-91803-5

**Published:** 2025-03-18

**Authors:** Mariama Souffou, Célia Dechavanne, Zaineb Kammoun, Firmine Viwami, Isabelle Gaugué, Naima Beldjoudi, Sébastien Dechavanne, Nawal Sare, André Garcia, Magalie Dambrun, Florence Migot-Nabias

**Affiliations:** 1 Université Paris Cité, MERIT, IRD, Inserm, Paris, Paris, F-75006 France; 2 Université Paris Cité, Cibles Thérapeutiques et Conception de Médicaments (CiTCoM), CNRS, Paris, France; 3 Epidemiology and Clinical Research Department, GH Paris Nord Val de Seine, Assistance Publique - Hôpitaux de Paris, Paris, France; 4 Centre d’Etude et de Recherche sur les Pathologies Associées à la Grossesse et à L’Enfance, Cotonou, Bénin; 5 Present Address: Genetics and Developmental Biology, Institut Curie, Université PSL, Sorbonne Université, CNRS UMR3215, INSERM U934, Paris, 75005 France

**Keywords:** Malaria, Parasitic infection

## Abstract

*Plasmodium falciparum* and *Toxoplasma gondii* are two apicomplexan parasites that can lead to severe complications for the newborn when contracted during pregnancy. This study explores the cross-reactivity of antibodies specific to both pathogens in pregnant women, exposed or not to malaria. The antibody response against full-length recombinant antigens from *P. falciparum* (*Pf*AMA1, *Pfs*48/45) and *T. gondii* (*Tg*AMA1, *Tg*SAG1, *Tg*GRA7), selected for their strong immunogenicity, was analysed on 150 plasma samples from women residing in Benin or France. The antibody functionality was assessed using *P. falciparum* in vitro Growth Inhibition Assay (GIA). As the main results, toxoplasmosis seropositive women with an ongoing *P. falciparum* infection better inhibited *P. falciparum* invasion compared to toxoplasmosis seronegative women (34.6% vs. 17.2%, *p* ≤ 0.01). Women with positive serologies for both parasites presented a significantly higher inhibition of *P. falciparum* invasion compared to those only seropositive for malaria (coef = 6.27, *p* = 0.076) in reference with double-negative women (coef = 11.35, *p* = 0.001). These data suggest that plasma samples containing anti-*T. gondii* IgG may contribute reducing the development of *P. falciparum* parasites. This study provides insight into the immune dynamics of the co-infection by these two apicomplexans with potential implications for developing cross-protective vaccines and therapies.

**Supplementary Information:**

The online version contains supplementary material available at 10.1038/s41598-025-91803-5.

## Introduction

The apicomplexans *Plasmodium falciparum (P.f)* and *Toxoplasma gondii (T.g*) remain the causal agents of two challenging parasitic human infections that affect large populations worldwide^1^. The harmfulness of both pathogens in a context of peri-natality, particularly in pregnant women, remains a major public health concern^2,3^. When these parasitic infections occur during pregnancy, the outcomes may be worsened because of hormonal and immunological adaptations aimed at protecting the foetus^4–7^.

In malaria during pregnancy, the affluent presence of *P.f-*infected red blood cells within the intervillous spaces of the placenta is associated with severe outcomes such as maternal anaemia, prematurity, or low birth weight^8–10^. The infected red blood cells within the placenta create an inflammatory setting that harms both placenta and foetus^11,12^. Although congenital malaria rarely occurs, it is secondary to the transplacental transmission of *Plasmodium*^13,14^. A primary infection with *P.f*typically induces an immune response characterized by the production of specific antibodies but the longevity of this immunity may vary considerably depending on the intensity and the duration of the infection^15,16^. Repeated exposure to the parasite contributes maintaining and reinforcing immunity over time^16^. However, when staying for a long time in settings where malaria transmission is low or absent, immune individuals may experience a gradual decline in antibody levels due to waning immune memory^17,18^.

On its side, congenital toxoplasmosis (CT) occurs in foetuses infected with the protozoan *T.g*^19^. The tachyzoite, which is the parasitic form capable of dissemination through the bloodstream, is directly transmitted from the primo-infected mother to the foetus and can cause miscarriage in the early pregnancy period. CT can also lead to progressive cognitive and visual impairments over the years in newborns from primo-infected pregnant women^20^. The immune antibody response to *T.g*is known to be robust and persistent. Indeed, the parasite can periodically reactivate from its dormant state, characterized by bradyzoites encysted in muscular and cerebral cells, into its circulating tachyzoite form with rapid replication^21–23^. The cyst persistence and antigenic stimulation due to re-infections contribute to the long lasting immune response to *T.g*^24^. The immune responses elicited against *P.f* and *T.g*exhibit distinct characteristics shaped by each parasite’s the unique biology which, in turn, can modulate the host’s immune system susceptibility^5,25^.

**Fig. 1:**
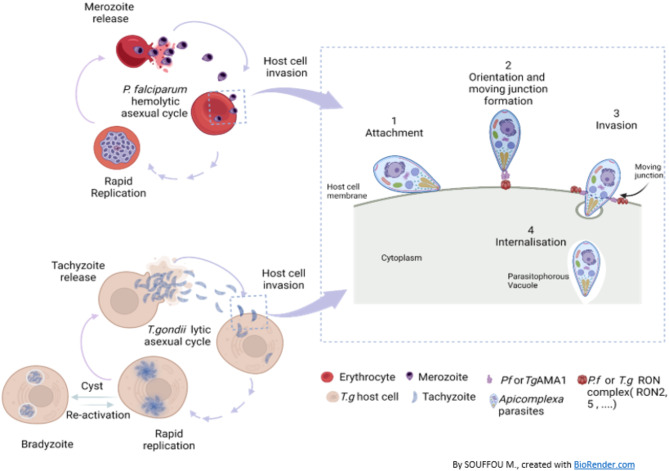
Illustration of the lytic asexual life cycle and the mechanisms of host cell invasion of the protozoan parasites *P. falciparum* and *T. gondii*. *P.f* host cells include only red blood cells. *T.g* host cells include macrophages, dendritic cells, muscle cells, neurons and other human cells. The illustration was created with BioRender.com .

**Fig. 2:**
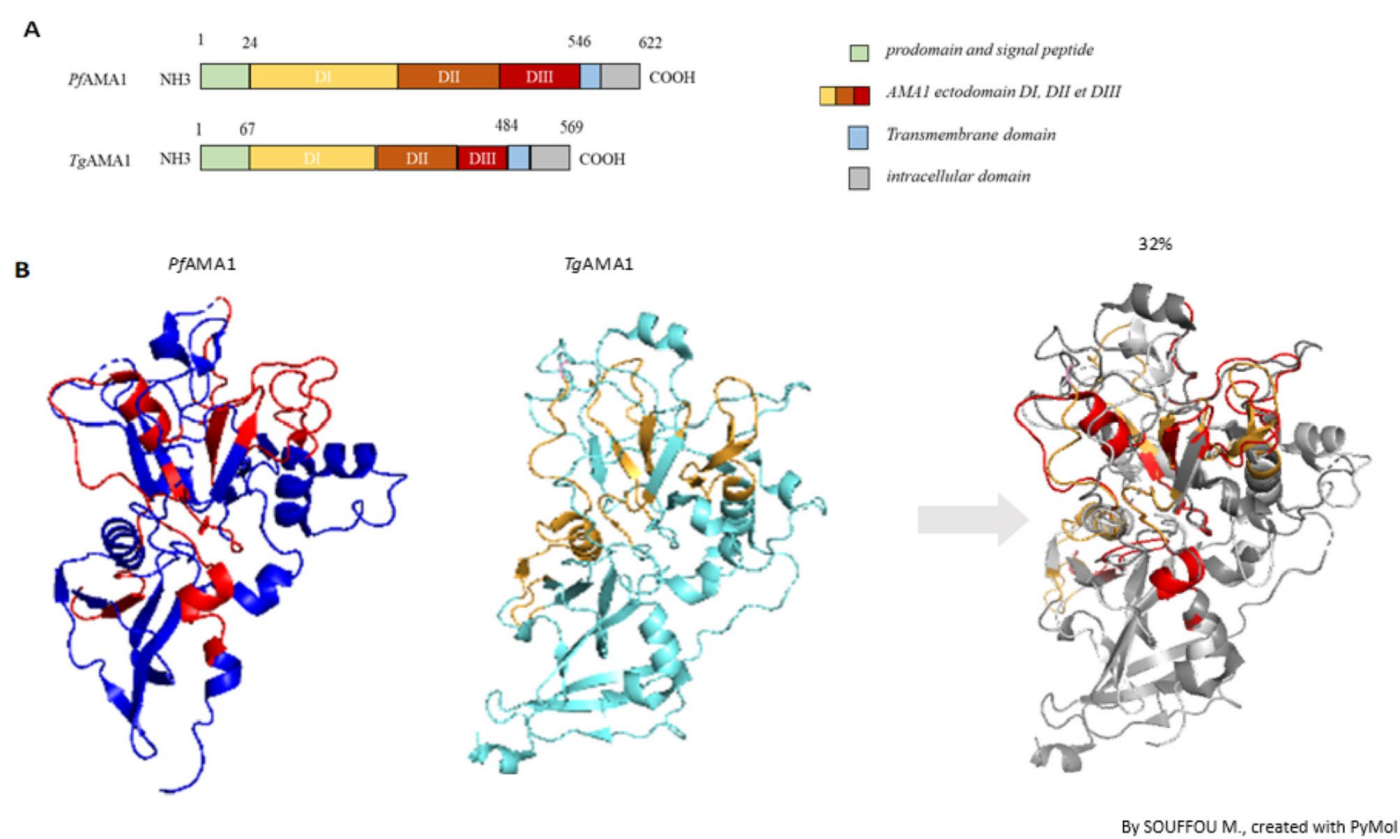
Structural similarities between *Pf*AMA1 and *Tg*AMA1. **A.** Illustration of the linear structure of *Pf*AMA1 and *Tg*AMA1 with the amino-acid positions delimiting the ectodomains. **B**. Three-dimensional structure of *Pf*AMA1 (PDB ref 4R19) in dark blue, *Tg*AMA1 (PDB ref 2X2Z) in cyan and their superposition using PyMOL software. Discontinuous predicted epitopes are indicated in red for *Pf*AMA1 and orange for *Tg*AMA1. RMSD = 2.63 Å; TM-score = 0.86. RMSD compares superpositions of three-dimensional protein structures, atom by atom. The closer this number is to 0, the closer is the structural alignment. RMSD has been associated with a protein structure alignment algorithm (TM-align) based on the TM-score^77^. This algorithm evaluates the quality of the aligned structures: 0.0 < TM-score < 0.30 = random structural similarity; 0.5 < TM-score < 1.00 = significant structural similarity.

Apicomplexan parasites share common biological mechanisms like the host cell invasion process involving the expression of similar proteins at their membrane surfaces^26,27^. It is the case of the apical membrane antigen (AMA1) protein and the rhoptry neck (RON) proteins complex (Fig. 1), particularly the RON2 protein^28,29^, which combination forms the moving junction and triggers the host cell invasion^28,30^. Both *Pf*AMA1 and *Tg*AMA1 antigens share structural similarities including a hydrophobic pocket that engage the interaction with the RON2 protein^31^ (Fig. 2A). This host cell invasion mechanism is well studied among apicomplexans for drug design or vaccine strategies^32,33^.

*Plasmodium* and *Toxoplasma* also share similar characteristics for the 6-cysteine protein superfamily (Supp. Figure 1)^34–36^. The most studied 6-cysteine antigens in malaria, are the *Pfs*48/45 gametocyte-specific antigens^37^eliciting antibodies that block parasite transmission to the mosquito vector^38,39^. In toxoplasmosis, the SAG1-related-sequence (SRS) family, including the major surface antigen SAG1, also adopts a 6-cysteine protein folding^37,40,41^. Experiments using anti-*Tg*SAG1 monoclonal antibody therapy in infected pregnant mice to reduce and prevent the vertical transmission of *T.g*to the offspring demonstrated the neutralizing role of these antibodies^42^.

These similarities between structure and function among apicomplexan proteins raise questions about common epitopes and immunomodulation in the context of pathogen-pathogen co-infection. By analogy with a mouse experimentation performed with *Plasmodium berghei*^43^, we propose that an already settled immunological environment of *T.g* infection may provide a supplementary protection against an acute infection with *Plasmodium*. Moreover, we have already shown that Beninese pregnant women seropositive for toxoplasmosis, as a reflect of a past infection, presented fewer *P.f* infections during their pregnancy than *T.g*seronegative women^44^. We also observed that among *P.f*-infected pregnant women, those who were seropositive for toxoplasmosis produced fewer specific antibodies against *Pf*AMA1 compared to toxoplasmosis seronegative women. Therefore, we hypothesized that anti-*T.g* IgG in seropositive women could interact with *P.f* antigens, preventing or slowing down the *Plasmodium* infection process. Cross-reactivity due to structural similarities was already described between *P. vivax Pvs*48/45 and *Pfs*48/45^45,46^ or between *Tg*AMA1 and *Neospora caninum Nc*AMA1^47^. Nevertheless, few data are described in the literature on *P.f*/*T.g*co-infection^48,49^, antigen similarities and natural acquisition of cross-reactive antibodies. Of course, such results may impact the choice of specific immunogenic antigens for the diagnosis of toxoplasmosis, considering that co-infection may lead to a cross-immune response as demonstrated for other apicomplexan species^50–52^.

The present study aims to provide a comprehensive exploration of the immunological intersections in *P.f* and *T.g* co-infections in the context of pregnancy. We attempted to provide an in-depth examination of the immune cross-reactivity resulting from the co-existence of both parasites in humans by means of structural modelling, epitope prediction followed by quantitative and qualitative assessments of specific IgG recognizing *Pf*AMA1, *Tg*AMA1, *Tg*SAG1, *Tg*GRA7 and *Pfs*48/45, in pregnant women either seropositive or seronegative for *P.f* and/or *T.g.*

## Results

### The bioinformatic analyses reveal conserved sites and predicted common B-epitopes

The study of the AMA1 linear sequences of the three ectodomains from *P.f* and *T.g* shows 32% identity at the amino acid level. This moderate level of similarity shows conserved sites in the sequence (Supp. Table 1). We aligned discontinuous B-cell epitopes along with conserved regions of *Pf*AMA1 and *Tg*AMA1 to identify possible common epitopes. The three-dimensional structures of *Pf*AMA1 and *Tg*AMA1 are remarkably close, with a root mean square deviation (RMSD) of 2,63 Å and a significant template modeling score (TM-score) of 0,86. The preserved 3D structures between both *P.f* and *T.g*AMA1 indicate that the two proteins have a similar key function, such as the binding on RON2 protein^26,28^. The structural alignment also reveals predicted common conformational B-epitopes within AMA1 (Fig. 2B). Conservation of common epitopes across *P.f* and *T.g* suggests potential cross-reactivity, where antibodies generated against *Tg*AMA1 might recognize, bind to, and even block the function of *Pf*AMA1 or vice versa.

**Table 1:** Univariate and multivariate linear regression analysis of factors associated with the functional antibody response.

Variables (*n*=147)^a^	Univariate		pval	Multivariate		pval
	coef ^b^	95% CI		coef ^b^	95% CI	
Serological status^c^ (reference: malaria non-exposed, TXD group)						
Malaria (+) and toxoplasmosis (−)	6.27	−0.68 ; 13.22	0.076			
Malaria (+) and toxoplasmosis (+)	11.35	4.48 ; 18.24	**0.001**			
Malaria (−) and toxoplasmosis (+)	0.42	−8.47 ; 9.30	0.926			
Toxoplasmosis seropositive	4.52	−0.39 ; 9.43	0.071			
Malaria seropositive	8.7	3.50 ; 13.94	**0.001**			
Weeks of amenhorrea	0.81	0.16 ; 1.47	**0.015**			
Age	−0.48	−0.91 ; −0.04	**0.031**			
IPTp doses^d^ (reference: 0 dose)						
1	−6.04	−18.91 ; 6.82	0.355			
2	−9.89	−22.00 ; 2.22	0.109			
3	−12.19	−24.06 ; −0.33	**0.044**			
Population groups^e^ (reference : TXD (France))						
COA (Benin)	9.36	3.22 ; 15.50	**0.003**	7.59	1.06 ; 14.12	**0.023**
PDC (Benin)	20.72	13.22 ; 28.21	**<0.001**	18.71	10.86 ; 26.57	**<0.001**
IgG anti-*Tg*AMA1	2.73	0.69 ; 4.77	**0.009**	2.00	0.06 ; 3.94	**0.043**
IgG anti-*Tg*AMA1 in category^f^ (reference: 0 (low))						
1 (medium)	3.88	−2.41 ; 10.16	0.225			
2 (high)	8.18	1.69 ; 14.67	**0.014**			
IgG anti-*Pf*AMA1	1.88	0.89 ; 2.86	**<0.001**			
IgG anti-*Pf*AMA1 in category^g^ (reference: 0 (low))						
1 (medium)	9.41	3.55 ; 15.28	**0.002**			
2 (high)	12.06	6.23 ; 17.90	**<0.001**			
IgG anti-*Tg*GRA7	2.18	−0.31 ; 4.69	0.085			
IgG anti-*Tg*SAG1	1.5	−0.13 ; 3.15	0.072			
IgG anti-*Pfs*48/45^h^	1.6	−0.44 ; 3.65	0.123			

^a^: n=147 including PDC (n=29), COA (n=95) and TXD (n=26 minus 3 individuals with missing data); ^b^: Coef= a positive coefficient indicates an increase of both independent and dependent variables. A negative coefficient indicates an increase of the independent variable while the dependent variable tends to decrease; ^c^: ref= malaria non-exposed (TXD group); ^d^: ref= zero IPTp dose received during the pregnancy follow-up; ^e^: ref= TXD group; ^f^: ref= individual with a lower response to *Tg*AMA1; g: ref= individual with a lower response to *Pf*AMA1; ^h^: the evaluation of antibody response against *Pfs*48/45 was only performed in COA (n=95) and TXD (n=26) groups.

### The three population groups display differences in their specific antibody responses

**Fig. 3:**
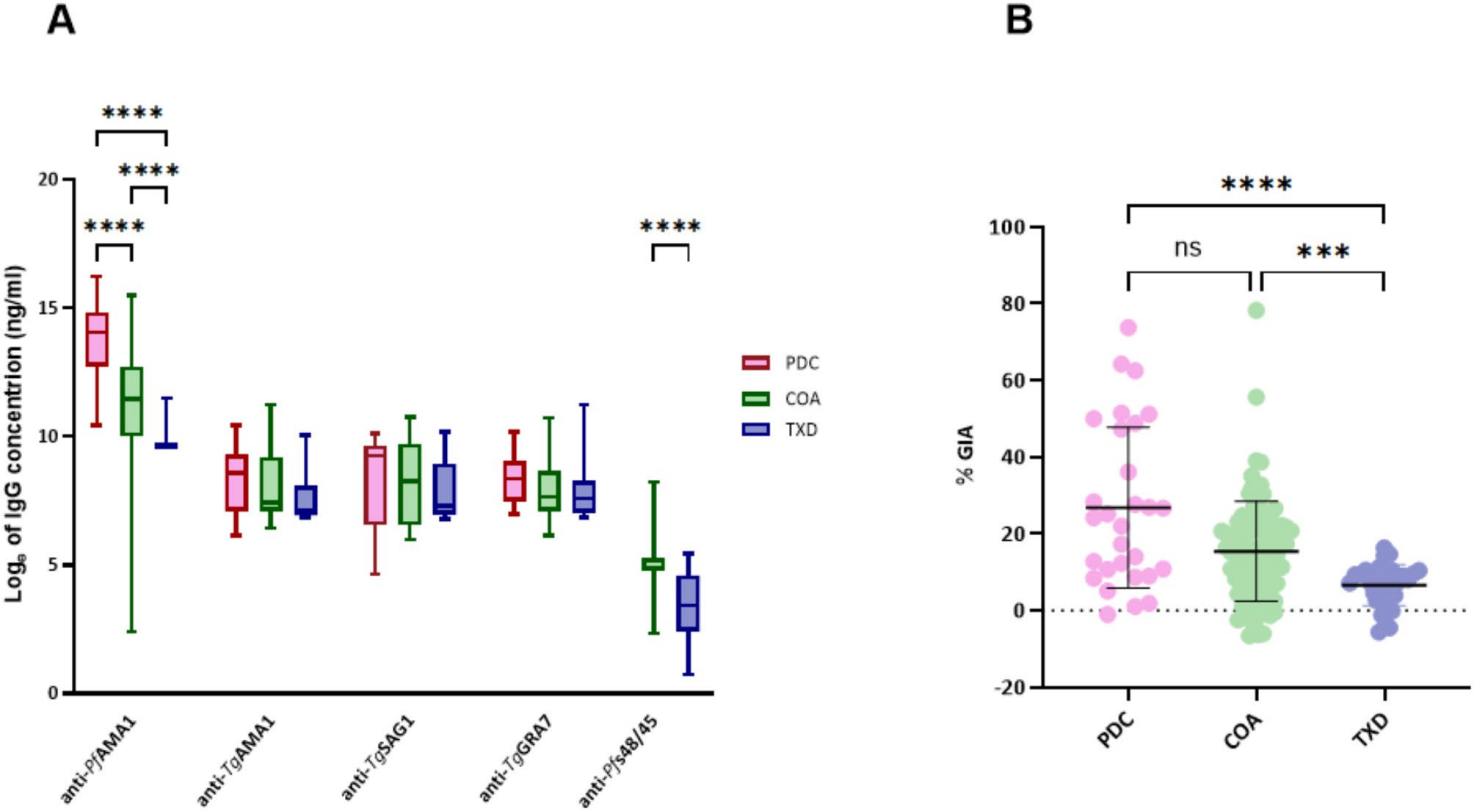
Description of the specific IgG responses in the three population groups. The PDC group (n =29) is represented in pink, the COA group (n =95) in green and the TXD group (n =26) in blue). **A.** The plot shows the logarithm of IgG concentrations (ng/ml) for each antigen tested (*Pf*AMA1, *Tg*AMA1, *Tg*SAG1, *Tg*GRA7, *Pfs*48/45) in the three groups. The box plots indicate the median and the whiskers represent the minimum and the maximum values. **B**. The scatter plots show the percentage of the *P.f* 3D7 growth inhibition (% GIA) for the three groups. Each dot represents an individual sample, with mean and standard deviation bars displayed. P-value significance p ≤0,05*, p ≤0,01**, p ≤0,001***, p ≤0.0001****.

In order to explore the antibody cross-reactivity against our recombinant antigens, we measured the specific IgG concentrations and levels of *P.f* 3D7-growth inhibition in the three population groups: PlasDCty (PDC, *n* = 29), CoaLa (COA, *n* = 95) and TOXODIAG (TXD, *n* = 26). Median IgG concentrations against *Pf*AMA1 ranged from 22.02 µg/ml [exp (10)] to 1202.60 µg/ml [exp (14)] across the groups while for IgG against *T.g* antigens (*Tg*AMA1, *Tg*SAG1 and *Tg*GRA7) the median concentrations were more clustered, varying from 1.09 µg/ml [exp (7)] to 8.10 µg/ml [exp (9)]. The lowest values of anti-*Pf*AMA1 and anti-*Pf*s48/45 IgG were observed for the malaria non-exposed TXD group comprising plasma samples collected in France, in comparison to the two Beninese groups exposed to malaria (PDC and COA) (Fig. 3A).

The PDC group, composed exclusively of women with an ongoing *P.f* infection, presented a wide range of its ability to inhibit the growth of the *P.f* 3D7 strain compared to the two other groups, thus indicating a variability in the individual immune effectiveness, combined with a high average response (Fig. 3B and Supp. Table 3). The COA group, comprised of plasma samples collected in pregnant women during the dry season in Benin, also displayed a wide variability in growth inhibition values. The TXD group, considered malaria non-exposed, presented the lowest values associated with less variability. These results highlight the groups’ quantitative and qualitative immune responses, depending on their characteristics such as the exposure to malaria parasites. In order to explore the IgG capability to cross-react, the toxoplasmosis serological status is considered more precisely in the following sections.

### The functional response against *P.f* infection is higher in the case of toxoplasmosis-positive serology

**Fig. 4:**
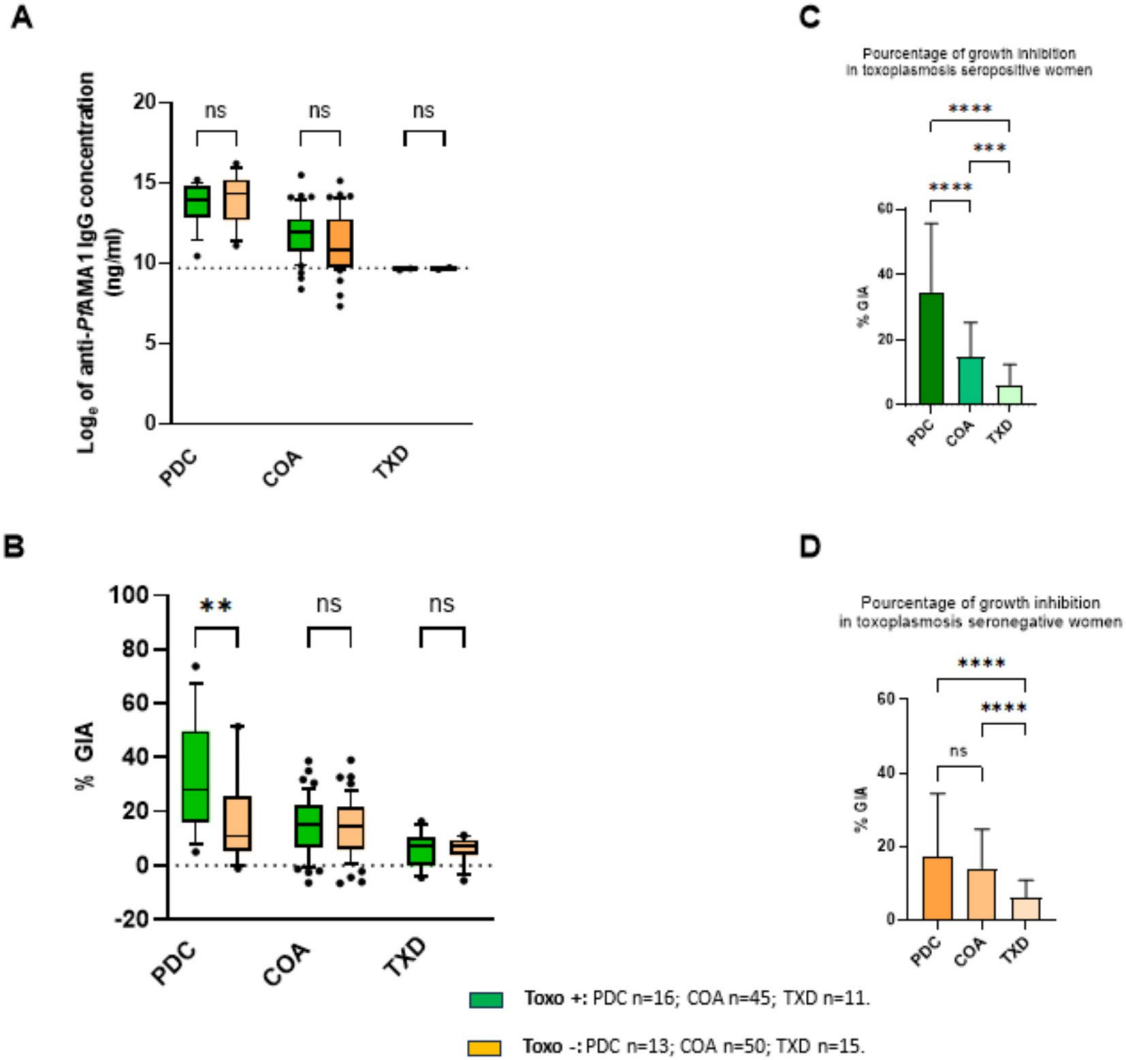
Quantitative and functional *P. falciparum* and *T. gondii* IgG responses in function of the toxoplasmosis serological status. Positive serological status is represented in green and negative serological status in orange. **A**: The levels of anti-*Pf*AMA1 IgG are compared between seropositive (Toxo+) and seronegative (Toxo-) toxoplasmosis women into each group (PDC, COA, TXD). The box plots indicate the median between 25^th^ and 75^th^ percentiles and the whiskers represent the 10^th^ and 90^th^ percentiles. The dots above the lines are outliers. **B** : In each group, the percentage of growth inhibition activity (% GIA) is compared between Toxo+ and Toxo- women. *P.f* growth inhibition activity is compared among Toxo+ (**C**) and Toxo- (**D**) women of each group. P-value significance p ≤0,05*, p ≤0,01**, p ≤0,001***, p ≤0.0001****. The histograms represent the means and standard deviations.

The IgG levels to *Pf*AMA1 did not vary significantly between toxoplasmosis seropositive (Toxo+) and seronegative (Toxo-) women in each group (Fig. 4A). Similarly, the IgG levels against *Pf*AMA1, *Tg*AMA1, *Tg*SAG1 or *Tg*GRA7 did not vary significantly among groups dichotomized according to their toxoplasmosis serological status (Supp. Figure 2). Meanwhile, PDC Toxo + women exhibited a stronger *P.f* growth inhibition activity compared to PDC Toxo- women (34.6% vs. 17.2%, *p* ≤ 0.01), which was not the case for the COA and TXD groups (Fig. 4B). Considering exclusively toxoplasmosis seropositivity, women in the *P.f*-infected PDC group presented higher GIA values than the malaria-exposed COA women (34.6% vs. 14.7%, *p* ≤ 0.0001) and malaria non-exposed TXD women (34.6% vs. 6.2%, *p* ≤ 0.0001) (Fig. 4C). Comparatively, the difference in growth inhibition efficacy between PDC and COA groups disappeared when considering only Toxo- women (Fig. 4D).

### The IgG response to *Tg*AMA1 is correlated to the IgG response to *Pf*AMA1 and to *P.f* growth Inhibition

**Fig. 5:**
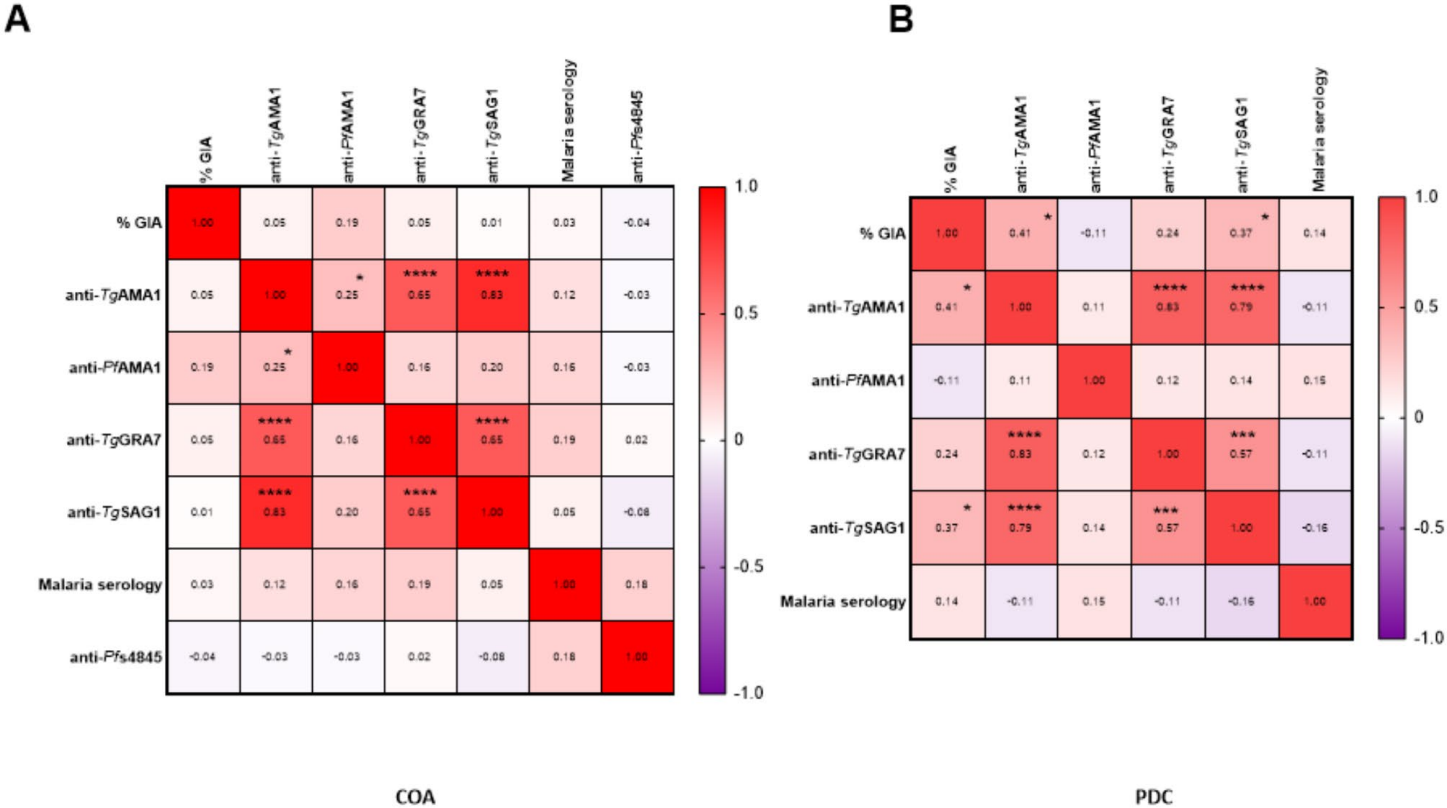
Correlations between anti*-T. gondii*, anti*-P. falciparum* antibody responses and *P. falciparum* growth inhibition data. The results are divided into two correlation heatmaps corresponding to the groups: **A**: CoaLa (n =95) and **B**: PlasDCty (n =29). The color scale indicates the strength of the Pearson correlation coefficient. Correlation coefficients and the degree of p-value significance ( p ≤ 0,05*, p ≤ 0,01**, p ≤ 0,001***, p ≤ 0,0001****) were displayed in each cell.

The analysis of correlations between antibody responses revealed the highest coefficients for IgG responses directed to *T.g* antigens when compared two by two (*Tg*AMA1/*Tg*GRA7, *Tg*AMA1/*Tg*SAG1, *Tg*GRA7/*Tg*SAG1). This result was observed for both COA and PDC groups (Fig. 5). *Tg*AMA1 IgG response was slightly positively related to *Pf*AMA1, only in the COA group (*r* = 0.25, *p* = 0.014). In addition, the anti-*Pf*AMA1 antibody response was weakly associated with the level of *P.f* 3D7 growth inhibition (*r* = 0.19, *p* = 0.059). Therefore, high levels of anti-*Pf*AMA1 IgG are not necessarily related to a strong inhibition activity, suggesting that not all secreted anti-*Pf*AMA1 IgG are functional. Anti-*Tg*AMA1 and anti-*Tg*SAG1 antibody responses in the PDC group were related to a higher *P.f* 3D7 growth inhibition activity (*r* = 0.41, *p* = 0,027 and *r* = 0.37, *p* = 0.048, respectively).

### IgG directed to *Tg*AMA1 are associated with *P.f* growth Inhibition

Univariate and multivariate analyses were conducted in order to further identify factors associated with the functional antibody response (Table 1). Univariate analysis revealed that women seropositive for malaria and toxoplasmosis exhibited higher inhibition (coef = 11.35; *p* = 0.001) than women only seropositive for malaria (coef = 6.27; *p* = 0.076). Specific IgG, particularly anti-*Tg*AMA1 (coef = 2.73; *p* = 0.009) and anti-*Pf*AMA1 (coef = 1.88; *p* < 0.001), showed significant positive associations with parasite growth inhibition. Multivariate analysis confirmed that anti-*Tg*AMA1 (coef = 2.00; *p* = 0.043), belonging to the COA group (coef = 7.59; *p* = 0.023) and more particularly to PDC group (coef = 18.71; *p* < 0.001), remained independently associated with the growth inhibition of *P.f* 3D7.

### Antibody responses to *P.f* and *T.g* are associated with toxoplasmosis serology

Not only IgG levels to *Tg*AMA1 (OR = 9.59; *p* < 0.001), *Tg*GRA7 (OR = 6.45; *p* < 0.001) and *Tg*SAG1 (OR = 40.56; *p* < 0.001), were strongly associated with a positive toxoplasmosis serology, but also IgG to *Pf*AMA1 (OR = 1.17; *p* = 0.034) (Table 2). Associations involving *Tg*AMA1 and *Pf*AMA1 remained in the multivariate analysis. Interestingly, the interaction between the IgG responses to *Tg*AMA1 and *Pf*AMA1, which represents the cross-reactivity between them, showed a negative association (OR = 0.59; *p* = 0.013), suggesting a moderate reduction effect of the toxoplasmosis serology positivity (Table 2).

**Table 2:** Univariate and multivariate logistic regression analysis of factors associated with toxoplasmosis positive serology.

Variables (*n*=148)^a^	Univariate		pval	Multivariate		pval
	OR^b^	95% CI		OR^b^	95% CI	
GIA	1.02	1.00 ; 1.04	0.076			
Population groups^c^ (reference: TXD group)						
COA (Benin)	1.31	0.55 ; 3.12	0.543	0.2	0.04 ; 0.96	**0.044**
PDC (Benin)	1.79	0.62 ; 5.17	0.282	0.41	0.05 ; 3.32	0.407
IgG anti-*Tg*AMA1	9.59	4.94 ; 18.64	**<0.001**	11172.85	35.65 ; 3,501,875	**0.001**
IgG anti-*Tg*AMA1 in category^d^ (reference: 0 (low))						
1 (medium)	6.56	1.80 ; 23.88	**0.004**			
2 (high)	133.16	27.97 ; 634.12	**<0.001**			
IgG anti-*Pf*AMA1	1.17	1.01 ; 1.36	**0.034**	47.25	2.16 ; 1034.94	**0.014**
IgG anti-*Pf*AMA1 in category^e^ (reference: 0 (low))						
1 (medium)	2.47	1.08 ; 5.61	**0.031**			
2 (high)	2.36	1.04 ; 5.34	**0.04**			
IgG anti-*Tg*GRA7	6.45	3.58 ; 11.61	**<0.001**			
IgG anti-*Tg*SAG1	40.56	8.92 ; 184.39	**<0.001**			
IgG anti-*Pf*s4548	1.02	0.74 ; 1.43	0.878			
IgG anti-*Tg*AMA1 x IgG anti-*Pf*AMA1				0.59	0.39 ; 0.89	**0.013**

^a^: n=148 including PDC (n=29), COA (n=95) and TXD (n=26 minus 2 individuals with missing data); ^b^: OR= an odds ratio >1 indicates that the event is more likely to increase as the independent variable increases. An OR <1 indicates that the event is less likely to occur as the independent variable increases; ^c^: ref= TXD group; ^d^: ref= individual with a lower response to *Tg*AMA1; ^e^: ref= individual with a lower response to *Pf*AMA1.

### Positive serology for toxoplasmosis and malaria enhances specific antibody responses

The analysis presented in Supp. Table 2 showed that women seropositive to both malaria and toxoplasmosis had higher antibody levels to *Pf*AMA1 (coef = 2.14; *p* < 0,001), *Tg*AMA1 (coef = 1.98; *p* < 0,001), *Tg*SAG1 (coef = 2.53; *p* < 0,001) and *Tg*GRA7 (coef = 1.41; *p* < 0,001) compared to the other serological groups. These outcomes indicate a strong immune response when the individual is immunized against both infections. However, for the antibody response against the gametocyte antigen *Pf*s48/45, the strongest association is related to the malaria seropositive group only (coef = 1.22; *p* < 0,001).

These findings highlight on one hand, the roles of the toxoplasmosis serological status and the immune malarial environment in terms of infection or exposure and on the other hand, the role of anti-*T.g* antibodies and more precisely the IgG, in reducing the development of *P.f* parasites. Pregnant women with a *P.f* ongoing infection (PDC group) presented a stronger association between IgG specific to *Tg*AMA1 and *P.f* growth inhibition activity than malaria-exposed non-infected women (COA group). The association between antibody responses to *T.g* and the functional activity of plasma antibodies against *P.f* parasite growth indicates a robust relationship between *T.g* and *P.f* immune responses in the context of *P.f* acute infection.

## Discussion

Our major findings suggest that an immunological context of toxoplasmosis may enhance the functional antibody response against *P.f*. These results could also imply differences in the immune response dynamics in *P.f* and *T.g* co-infection. Data from Beninese women belonging to the PDC group, composed exclusively of women with a confirmed *P.f* infection, and to the COA group, including women exposed to malaria without necessarily presenting an ongoing infection with *Plasmodium*, were compared to those of non-malaria exposed women from the TXD group in France. Our results allowed us to demonstrate the importance of exposure and infection status regarding malaria on the qualitative aspects of the immune response directed against the two pathogens.

Besides, population-specific differences were evident, with the PDC and COA groups exhibiting significantly higher capacity of *P.f*growth inhibition than TXD. Environmental factors, such as residence in Benin and the rainy season, were associated with a higher inhibition capacity, due to a related increase in plasmodial infections compared with the dry season. Malaria transmission follows a seasonal pattern that may be related to the characteristics of the COA and PDC cohorts. Indeed, these findings align with research done on malaria seasonality in Cotonou in southern Benin, which demonstrated a low incidence among children in March-April at the end of the long dry season and a peak of the malaria incidence in November-January following the end of the short rainy season^53^. A 2021 study by Filémon et *al.*also observed minimal malaria transmission in December-February during the long dry season^54^.

Moreover, the linear and structural alignments of the proteins *Pf*AMA1 and *Tg*AMA1 involved in the invasion by *P.f* and *T.g*, of their respective host cells, have shown significant similarities. The two proteins exhibit a high linear sequence similarity as well as a remarkably close three-dimensional structure. The conservation between the two *Apicomplexa*proteins is probably due to their common function^26^. Indeed, this degree of conservation is critical as it indicates that both proteins adopt a similar three-dimensional structure, allowing them to perform analogous functions in both *Plasmodium* and *Toxoplasma*parasites, like binding to the RON2 protein^27,28^. The presence of predicted common epitopes within AMA1 sequences suggests potential cross-reactivity, in some conserved areas, as it is known that 90% of the B cell epitopes are discontinuous^55^. It means that antibodies generated against *Tg*AMA1 might recognize and bind to *Pf*AMA1 and vice versa in in vitro experiments. We have voluntarily chosen to produce the antigens as full-length proteins to be as close as possible to the biological conformation of the proteins. However, conserved peptides were not tested individually to validate a cross-reactive region. A recently published study by Najm et *al.*in 2023^56^ demonstrated the importance of the in vivo complex of AMA1-RON2 to generate a complete immune response compared to AMA1 alone.

The analysis of antibody levels against *P.f* and *T.g* antigens across three population groups (PDC, COA, TXD) indicated no significant differences between anti-*Tg*AMA1 and anti-*Pf*AMA1 IgG levels in toxoplasmosis seropositive pregnant women but also in seronegative ones. We can suppose that the level of cross-reactive IgG is too residual to be quantitatively evaluated with a direct method. Indeed, measuring a significant increase in *T.g*-specific IgG levels with indirect ELISA is challenging because the IgG response to *T.g* antigens, like *Tg*AMA1, is much weaker than the response to *P.f* antigens, such as *Pf*AMA1. The detection cut-offs also vary between these antigens, making comparisons difficult. Additionally, some antibodies might cross-react between *T.g* and *P.f* antigens, which could reduce the apparent *T.g*-specific response by being overshadowed by the stronger malaria-specific IgG. We combined the quantitative ELISA results with a functional test to address these issues. This test allowed for a better understanding of the antibody activity, giving a more accurate overall result. Furthermore, in univariate analysis, anti-*Tg*AMA1 IgG along with anti-*Tg*GRA7 and anti-*Tg*SAG1 IgG have emerged as robust predictors of toxoplasmosis positivity, with *Tg*SAG1 as the strongest one, suggesting their critical role as biomarkers for a toxoplasmosis infection. Indeed, the three *T.g*recombinant antigens concerned in this study are considered in the literature for their immunogenic potential in the diagnosis of acute and chronic toxoplasmosis infection^57–59^.

Conversely to quantitative IgG results, the results of the IgG functionality through the *P.f* growth inhibition measured by GIA revealed different outcomes. Indeed, toxoplasmosis seropositive women with an ongoing *P.f* infection (PDC) presented the highest inhibition percentages, in comparison to the malaria-exposed group (COA). Those findings indicate a robust functional *P.f* antibody response in toxoplasmosis seropositive and *P.f*-infected women. Conversely, no significant differences were observed in the antibody functionality among toxoplasmosis seronegative women in both cohorts. Even if malaria seropositivity alone tended to be associated with *P.f* parasite inhibition, malaria and toxoplasmosis seropositive women exhibited a better association with the inhibition of *P.f* 3D7, reflecting a strong immune response against the development of *P.f* parasites. Anti-*Pf*AMA1 IgG are widely reported in the literature as inhibitory antibodies that prevent *P. falciparum*invasion of red blood cells^60^. The synergy between anti-*Tg*AMA1 and anti-*Pf*AMA1 IgG suggests a complex interplay in co-infected individuals, indicating that their joint presence might influence the development of *P.f* parasites in an infected host differently than when present alone. However, IgG directed against the homologous *Pfs*48/45 and *Tg*SAG1 antigens were not significantly associated with the inhibition of the parasite. An explanation might be that anti-*Pfs*48/45 IgG are *P.f*gametocyte inhibitors and therefore are not specific to merozoites^39^.

An experiment led by Omata et al.. in 1981^48^ has demonstrated that mice immunized against tachyzoites had developed an immune resistance against plasmodial infection (*Plasmodium berghei)*. However, the reciprocity is not confirmed, as *Plasmodium*-infected mice did not show a protective reaction against *T.g*tachyzoites (RH strain) inoculation^48^. Another study, published by Ndamukong-Nyanga et al.^49^, has suggested that toxoplasmosis seropositive pregnant women in Cameroon were more resistant to infections with *Plasmodium*. However, on the opposite, the severity of the toxoplasmosis infection was not related to the malaria serological status. These findings are in accordance with our previous study (Dambrun et al.)^44^, where we demonstrated that Beninese pregnant women with a toxoplasmosis seropositive status had fewer *P.f* infections than seronegative women. The present results lead to the hypothesis that mechanisms of protection against *P.f* infection could involve a large scale of immune responses to *P.f* and *T.g* apicomplexan parasite infections. The most probable hypothesis to explain the boost of the IgG functionality against *P.f*infection in toxoplasmosis seropositive women involves the T-cell response. Indeed, the existence of cross-reactive T-cells has been demonstrated between diseases such as influenza and COVID^61^. The T-cells display a large repertoire of antigenic peptides, with a T-cell receptor possibly recognizing multiple peptides as described by Sewell^62^. The study of Su et al. demonstrated that memory T-cells from an adult non-exposed to a specific viral pathogen can recognize some specific viral antigens because of the microbial environment to which the individual had been exposed^63^. Regarding this information, we can suppose that the development of malaria could activate cross-reactive memory T-cells related to particular antigens of *T.g*, like *Tg*AMA1, and enhance the functional antibody response against malaria.

Meanwhile, several investigations have found that, while infected patients can produce an initial immune response, antibody levels drop rapidly in the absence of *Plasmodium* re-exposure, mainly due to the decline in antibody-secreting cells. Repeated exposure to *Plasmodium*helps to maintain or even extend the duration of these^15,16^. It is known that IgG1 and IgG3, also referred to as cytophilic, can recruit macrophages, neutrophils and eosinophils by anchoring their Fc fragment to the membrane receptors for Fc (FcγR) present on these cells. They are the main subclasses involved in the humoral response against *P. falciparum*^64,65^. IgG1 and IgG3 can also activate the classical complement pathway by binding to the C1q protein, forming the Membrane Attack Complex (MAC), which results in the lysis of infected cells or circulating parasites.

In contrast to *P. falciparum*, the humoral response against toxoplasmosis is known to be persistent throughout life^66^. Indeed, the presence of cysts composed of bradyzoites in the smooth tissues of the host induces regular antigenic stimulation, which contributes to long-term immunity against *T. gondii*^24^. IgG1 and IgG3 are also the main immunoglobulins secreted during the humoral response to the antigenic proteins of *T. gondii*, such as SAG1^67^. These immunoglobulins opsonise tachyzoites, allowing macrophages and other phagocytic cells to quickly eliminate circulating parasites^68^. As in the case of *P. falciparum*, they can activate the complement system, causing infected cells to lyse. Despite the pertinence of our outcomes, these results should be handled carefully considering the differences in the designs of the three population groups studied and the small sample size of the PDC and TXD groups. We used plasma samples containing Fc-dependent IgG antibodies for the growth inhibition assay instead of purified antibodies. It means that other non-specific cross-reactive antibodies in the plasma might have influenced the results, which could affect the specificity of our findings. Moreover, the cross-reactivity study was performed in vitro with refolded recombinant proteins and could potentially not reflect precisely the biological process expected. We also have to consider that *Pf*AMA1 is a polymorphic antigen and our study was conducted with a laboratory *P.f* strain (3D7), which does not reflect the dynamics of field-circulating *P.f* isolates. The GIA was performed using the 3D7 *P.f* strain, even if the protective effect tends to be procured by *T.g* antibodies, the reciprocation cannot be verified without further experiments using *T. gondii* tachyzoites. Thus, other factors like individual genetic differences should be considered in further studies.

## Conclusion

The in silico results combined with the experimental study of *P.f* and *T.g* antibody responses indicate a potential cross-reactivity between *P.f* and *T.g*. The hypothesis that a settled *T.g* infection could confer protection against *P.f* infection is supported by the observed enhancement in functional antibody responses among toxoplasmosis seropositive women, helping the clearance of the *P.f* parasite and preventing clinical complications of a *P.f* infection. Indeed, studies with plasmas from pregnant women indicate that toxoplasmosis may confer a protective effect against malaria, potentially through antibody-mediated or cellular immune mechanisms. This cross-reactivity might modulate the immune response, offering insights into protective mechanisms. Further research is needed to elucidate the underlying biological processes and reinforce our results. These findings could have significant implications for the development of cross-protective vaccines and therapies.

## Materials and methods

### Biological samples

The study was performed with plasma samples obtained from pregnant women differentially exposed to *P.f* and/or *T.g* parasites in order to evaluate the joint antibody response to both pathogens. For this purpose, 150 plasmas, all preserved at −20 °C until use and originating from three different field studies, were selected (Table 3).

**Table 3:** Characteristics of the 3 population groups of pregnant women under study.

	Year	Season and residence	Time of sampling	Exposure to *Plasmodium*	Malaria seropositive	Malaria seronegative	Toxoplasmosis seropositive	Toxoplasmosis seronegative
**PlasDCty - PDC** (*n* = 29)	2021	Rainy season Akassato, Benin	Delivery	Exposed, *Plasmodium* infected ^a^	28 (97%)	1 (3%)	16 (55%)	13 (45%)
**CoaLa - COA** (*n* = 95)	2018	Dry season Cotonou, Benin	Last prenatal visit of the 3^rd^ trimester	Exposed, Infectious status unknown	79 (83%)	16 (17%)	45 (47%)	50 (53%)
**TOXODIAG - TXD** (*n* = 26)	2018 to 2023	_ Île de France, France	Delivery	Non-exposed	0 (0%)	26 (100%)	11 (42%)	15 (58%)

^a^: PCR and/or RDT malaria positive

#### PlasDCty samples (Benin)

The PlasDCty (PDC) study took place from June to November 2021 in the maternity of Akassato. This locality in southern Benin (6° 30’ 0” North, 2° 22’ 0” East) is located in the Atlantic Department, 20 km northwest of Cotonou, the economic capital of Benin. The climate in this area is sub-equatorial, with two rainy seasons extending from April to July and October to November. Malaria is perennial, with transmission peaks during the rainy seasons. *Anopheles gambiae* and *An. funestus* are the main malaria vectors, and *Plasmodium falciparum*is the major infecting species^69^. The study was therefore conducted over the two rainy seasons. It was designed to evaluate the immune modulation of neonatal dendritic cells (DC) due to maternal infection and their ability to activate T cells to design a potential vaccine candidate able to fully activate the DC. Women were screened at delivery (*n* = 337) for malaria using *Pf*HRP-II and *Plasmodium* LDH Rapid Diagnosis Test (RDT) to select all 29 pregnant women with confirmed *P.f* infection, based on microscopic and/or PCR results. Regarding microscopic examination, parasites and peripheral blood mononuclear cells were counted on 10% Giemsa-stained thick blood smears up to 500 leucocytes. If no parasite was detected, an extra 500 leucocytes were counted to ensure the negativity. Parasitemia was estimated using 8000 leucocytes per microliter of blood following the formula: parasites/µL blood = (No. of parasites counted x 8000 white blood cells/µL) / No. of white blood cells counted. For PCR, DNA was extracted from 200 µL of whole blood using DNeasy Blood kit (Qiagen, France) and used to detect *Plasmodium* species (*P. falciparum*, *P. malariae* and *P. ovale*) by nested PCR amplification of the 18s small subunit ribosomal RNA gene as previously published^70^. Inclusion criteria were women of 18 years old or older, a priori termed delivery with expected non-complication. Diagnostic testing with Platelia™ Toxo IgG (Bio-Rad, France) and Malaria EIA (BioRad, France) kits was performed to determine each woman’s toxoplasmosis and malaria serological status.

#### CoaLa samples (Benin)

The CoaLa (COA) study was carried out from February to early April 2018 at the maternity ward of the CHU-MEL (Centre Hospitalier et Universitaire de la Mère et de l’Enfant Lagune), located in the center of Cotonou, in the Littoral Department of Benin. Most women admitted to this hospital, specialized in gynecology, obstetrics and pediatrics, reside in Cotonou or the surrounding area. The climate is similar to what was described above, so the study occurred during the dry season, corresponding to low or no malaria pressure. It was designed as part of the implementation of a new diagnostic approach for congenital toxoplasmosis and concerned 106 women recruited at their last antenatal care visit, in their third trimester of pregnancy^71^. Inclusion criteria were: at least 18 years of age and pregnancy 7 months, clinical uncomplicated delivery planned at the maternity, and residing in neighborhoods close to CHU-MEL. A total of 95 plasma samples were available for the present study. Toxoplasmosis serology was performed with *recom*Well *Toxoplasma* IgG kits (Mikrogen Diagnostik, Germany) and verified by VIDAS^®^ TOXO IgG II (bioMérieux, France) and malaria serological status was determined with Malaria EIA (Bio-Rad, France) kits.

#### TOXODIAG samples (France)

The TOXODIAG (TXD) study was carried out between 2018 and 2023 among 60 women performing prenatal follow-up and giving birth in the maternity wards of 5 hospitals from the AP-HP (Assistance Publique-Hôpitaux de Paris, France). Inclusion criteria were at least age 18 years and clinically uncomplicated delivery. The study aimed to identify women diagnosed with toxoplasma seroconversion during pregnancy as part of the implementation of a new diagnostic approach for congenital toxoplasmosis. Among the available collected samples during the study, 26 plasma samples corresponding to malaria non-exposed women were selected and divided into two groups according to the serological toxoplasmosis results delivered by the hospital biology laboratories. The malaria serological status was performed with Malaria EIA (Bio-Rad, France) kits in order to check that the women were seronegative for malaria.

### Sequence analysis and structure prediction with bioinformatic tools

#### Sequence homology analysis

A Basic Local Alignment Search Tool (BLAST) was performed to identify homologies in public databases. This was followed by a global alignment using the MUSCLE program in MEGA X software (https://www.megasoftware.net/) to determine sequence similarities between *P.f* and *T.g* proteins targeting *Pf*AMA1 and *Tg*AMA1. The Vector Alignment Search Tool (VAST+) was used to identify structural similarities between *P.f* and *T.g* proteins.

#### Epitope prediction tools

An epitope prediction mapping was implemented by using the Immune Epitope Database (IEDB) Analysis Resource to understand antigen-antibody interactions. The database provides a comprehensive suite of bioinformatics tools for B-cell and T-cell epitope prediction. Conformational/discontinuous B-cell epitopes resulting from 3D protein structures (Protein Data Bank) were explored with the IEDB Disco Tope tool (Version 1.1) associated with CB Tope and BCPREDS servers for a robust selection of recurrent predicted epitopes. The default parameter was selected for the threshold. Structural analyses were handled by the PyMol software 3.0. Common B-cell epitopes were identified on the aligned *Pf*AMA1 et *Tg*AMA1 protein sequences.

#### Design of Recombinant antigens and expression vectors

The analysis of the protein sequences and the secondary structure prediction was established using ExPASy tools resources and particularly the TMHMM to predict transmembrane domains and signal peptides. The designed pET28a + expression vector, including selecting suitable restriction sites, was verified with Serial Cloner (Version 2.6.1).

### Production of *Pf*AMA1, *Tg*AMA1, *Tg*SAG1 and *Tg*GRA7 recombinant antigens

#### Transformation and expression of the recombinant proteins in *E. coli*

The *Pf*AMA1 sequence (Q7KQK5) was selected from the 3D7 strain in the PlasmoDB database. The 3D7 strain is a clone from the NF54 isolate, which originates from African regions^72^. 3D7 is a reference laboratory strain for in vitro drug testing and inhibition assays^73^. The genetic sequences of the selected recombinant antigens *Tg*AMA1 (B6KAM0), *Tg*SAG1 (A0A125YP09) and *Tg*GRA7 (A0A125YI90) were identified in the ToxoDB database, referencing the type II Me49 strain. Type II strain was prioritized because of its worldwide dissemination notably in Europe and Africa and also for its implication in CT^74,75^. Each antigen’s coding sequences were synthesized and fused to a C-terminal 6 histidine-tag inserted into a pET28a + expression vector (Genscript, Piscataway, Netherlands). The transformation was carried out using competent SHuffle bacteria (T7 *E. coli*) for the expression of *Pf*AMA1 (24-546aa) and *Tg*AMA1 (67-484aa), and *E. coli* BL21C + for the expression of *Tg*SAG1 (46–336 aa) and *Tg*GRA7 (27-183aa). Our recombinant proteins were produced in full length, excluding only the signal sequences and the transmembrane domains associated with intracellular peptides as annotated by UniProt.

The bacterial transformation was performed following the Neb Bioland protocol described on the website (https://www.neb.com/en/protocols/2012/05/21/transformation-protocol). Transformed bacteria were spread on a Petri dish containing LB agar (Sigma Aldrich, St-Quentin-Fallavier, France) supplemented with 25 µg/ml of kanamycin (Gibco, United Kingdom). The plate was then incubated for 24 h at 30°C for transformed SHuffle bacteria and 37°C for BL21C + to allow bacterial growth and selection.

#### Bacterial culture and induction

A single colony from the selection plate was transferred to 25 mL of Luria Broth medium (Invitrogen™, France) supplemented with 25 µg/ml of kanamycin. The pre-culture was incubated overnight at 30 °C for SHuffle bacteria and 37°C for BL21C+. Bacteria were then cultured in 1 L of Luria Broth medium containing 25–50 µg/ml of kanamycin, with a starting optical density (OD) of 0.1. Protein expression was induced for 3 h by adding 1 mM of isopropyl β-D-1-thiogalactopyranoside (IPTG) (Sigma Aldrich, St-Quentin-Fallavier, France) when the bacterial growth reached an OD between 0.6 and 0.8. The bacterial pellet was collected by centrifugation at 4000 rpm for 30 min and stored at −20°C for later use.

An SDS-PAGE gel (Bio-Rad, France) was performed to confirm protein induction at the expected molecular weight for each antigen i.e. 75 kDa for *Pf*AMA1, 50 kDa for *Tg*AMA1, 30 kDa for *Tg*SAG1 and 25 kDa for *Tg*GRA7 (Supp. Figure 3 and Supp. Figure 4).

#### Purification of *Pf*AMA1, *Tg*AMA1 and *Tg*SAG1 recombinant proteins

The bacterial cells were lysed in phosphate buffer saline (Gibco™ PBS, Fisher Scientific, France) lysis buffer with the NaCl concentration adjusted at 350 mM, supplemented with 1 tablet of protease inhibitor cocktail (Roche cOmplete™ EDTA free), 5 mM DTT and 100 mM of lysozyme (all products from Sigma Aldrich, St-Quentin-Fallavier, France). The suspension was incubated on ice for 15 min and then sonicated six times alternating 10-second cycles of sonication and rest, to disrupt cell membranes and release the proteins expressed in inclusion body (IB) forms. The sonicated lysate was centrifuged at 6000 rpm, at 4°C for 30 min. The supernatant was discarded and the pellet, containing the IB, was washed twice with lysis buffer supplemented with 4 M urea (Fisher Scientific, France) to remove impurities. The IB were then dissolved overnight at 4°C in a buffer at pH 8 containing PBS 1X, 1% Triton X-100 (Fisher Scientific, France) and 6 M urea. The dissolved mixture was centrifuged at 9000 rpm for 30 min at 4°C to remove insoluble debris. The solubilized proteins were incubated with Ni-NTA agarose resin (Fisher Scientific, France) at 4°C for 1.5 h for metal affinity purification of the 6xHis-tagged recombinant fusion proteins. The protein refolding was initiated by gradually reducing the urea concentration from 6 M to 4 M and 2 M until reaching a final concentration of 1 M.

The *Pf*AMA1 recombinant protein was refolded on Ni-NTA column in PBS 1X buffer at pH 8.5, supplemented with 5 mM of reduced glutathione GSH and 0.5 mM of oxidized glutathione GSSG (Sigma Aldrich, St-Quentin-Fallavier, France) for 2 h at 4°C. The refolded protein was then eluted in PBS 1X with 400 Mm of imidazole (Arcos Organics, Geel, Belgium). In order to remove the residual contaminants, the eluate was filtrated in size chromatography by injecting 5 mL of the eluted protein into the gel filtration column (HiLoad^®^ 16/600 Superdex^®^ 200, Cytiva) connected to an Äkta purifier system (AKTA pure™, Cytiva).

The *Tg*AMA1 and *Tg*SAG1 recombinant proteins were eluted in PBS 1X with 1 M urea and 400 mM imidazole. The proteins were then dialyzed against a 10 kDa cut-off membrane (Slide-A-Lyzer™ G3 Dialysis Cassettes, Fisher Scientific, France) for 24 h in PBS 1X buffer at pH 8.5, supplemented with 3 mM GSH and 0.3 mM GSSG.

The concentrations of the three purified proteins were measured at 280 nm using a Nanodrop and aliquots were stored in glycerol 10% at −80°C for long-term preservation.

#### Purification of the soluble TgGRA7 Recombinant protein

The soluble GRA7 protein was directly purified on the Ni-NTA resin from the supernatant obtained after the lysis of the bacterial cells. The protein was subsequently eluted from the nickel column using 400 mM imidazole after three washes with PBS 1X containing 20 mM imidazole. Gel filtration was then performed to remove *E. coli* contaminants. The eluted protein was concentrated before being injected into a gel filtration column connected to an Äkta system. The protein concentration was measured at 280 nm using a Nanodrop and aliquots were stored in 15% of glycerol at −80°C for long-term preservation.

#### Pfs48/45 Recombinant antigen

The gametocyte recombinant antigen *Pf*s48/45 was produced and kindly gifted by Michael Theisen laboratory research^76^ (Department for Congenital Disorders, Statens Serum Institut, Copenhagen, Denmark).

### Titration of specific IgG directed to *Pf*AMA1, *Tg*AMA1 *Tg*SAG1 and *Tg*GRA7 with an enzyme-linked immunosorbent assay (ELISA)

Nunc MaxiSorp 96-well microtiter plates (Thermo Scientific™, France) were coated with purified IgG sourced from human serum (Sigma Aldrich, St-Quentin-Fallavier, France) at concentrations ranging from 5000 to 10 ng/ml, in PBS 1X coating buffer at pH 8.5. Additionally, recombinant antigens were individually coated at a concentration of 1.5 µg/ml for *Pf*AMA1, *Tg*AMA1, *Tg*SAG1 and *Tg*GRA7 and of 0.5 µg/ml for *Pfs*48/45 in coating buffer, with 100 µl added per well, and left to incubate at 4 °C overnight. Plasma samples were appropriately diluted (1:5000 for *Pf*AMA1 and 1:100 for *Tg*AMA1, *Tg*SAG1, *Tg*GRA7 and *Pf*s48/45 antigens) in dilution buffer [PBS 1X − 0.5% Tween 20 − 1% BSA (Sigma Aldrich, St-Quentin-Fallavier, France) − 0.02% sodium azide (Sigma Aldrich, St-Quentin-Fallavier, France)]. Following the dilution, the coated plates underwent three washes of 1 min each with washing buffer [PBS 1X − 0.5% Tween 20 − 0.5 M NaCl]. Subsequently, each well received 150 µl of blocking buffer [PBS 1X − 0.5%Tween 20 − 3% BSA], and the plates were then incubated at room temperature for 1 h. The diluted plasma samples (100 µl each) were added in duplicate to the coated plates containing the recombinant antigens, and then incubated for 1 h. After another round of washing, a primary antibody (mouse anti-human Fc, Sigma Aldrich, Schnelldorf, Germany) diluted at 1:4000 in dilution buffer was applied and left to incubate for 1 h at room temperature on a rocking platform, followed by three washes with washing buffer. Finally, a secondary antibody (peroxidase-conjugated goat anti-human IgG H + L, Bio Rad, France) was added at 1:6000 in dilution buffer. After the last wash, 100 µl of 3,3′,5,5′-Tetramethylbenzidine (TMB, Sigma Aldrich, St-Quentin-Fallavier, France) were added to each well and incubated for 30 min at room temperature in the dark. The reaction was stopped by adding an equal volume of 0.2 M sulfuric acid (H2SO4, Sigma Aldrich, St-Quentin-Fallavier, France). Absorbance was read at 450 nm with a reference wavelength of 620 nm using a plate reader (TECAN Infinite 50). The concentration of specific antibodies was calculated using MyAssay Software (https://www.myassays.com/) using a five-parameter logistic modelisation. For each antigen, the cut-off was represented by calculating the mean + 2 SD of the values obtained in the group of 15 malaria non-exposed and toxoplasmosis seronegative plasma samples from the TOXODIAG study.

### *In vitro *growth inhibition assay (GIA) of merozoites from the 3D7 strain of *P. falciparum*

To assess the *P.f* inhibitory activity of the anti-*T.g* antibodies contained in the plasma samples, an in vitro culture of the *P.f* 3D7 strain was maintained with O + red blood cells from healthy donors at a 5% haematocrit in T75 sterile culture flasks containing 25 ml of RPMI 1640 medium (Gibco™, Fisher Scientific, France) – 25 mM HEPES (Gibco™, Fisher Scientific, France) at pH 7.4 supplemented with 20 mg/ml hypoxanthine (Gibco™, Fisher Scientific, France), 25 mM NaHCO3 (Fisher Scientific, France), 2.5 mg/ml gentamycin (Gibco™, Fisher Scientific, France), 0.5% Albumax II (Gibco™, Fisher Scientific, France) and 10% human AB serum (Biowest, France) in an atmosphere of 5% O2, 5% CO2 and 90% N2. The culture medium was changed every 24 h while maintaining a parasitemia below 5%. Thin blood smears were made to verify the parasite stages and calculate parasitemia. Parasite stages were synchronized and parasites were concentrated using a magnetic activated cell sorter (MACS) column (Miltenyi Biotec, Germany) to collect schizont forms. These mature forms were diluted at a 2% haematocrit in the culture media previously described, at an initial parasitaemia of 1% for one-cycle assays. For multi-plate assays, a malaria-positive control plasma (PC) selected from the PDC group with a high inhibition activity and seronegative for toxoplasmosis was included in each plate along with a pool of negative control plasmas (NC) collected from 10 French donors non-immune against malaria and toxoplasmosis (National Malaria Reference Center, Bichat - Claude Bernard Hospital). The assay setup involved adding 100 µl of the suspension of *P.f* 3D7 mature forms to each well of 96-well culture plates, adjusting the plasma dilution to 1:50 final concentration. Each sample was tested in triplicate. The plates were maintained in a specialized culture chamber fully gassed at 5% CO_2_ before incubation at 37 °C and 90% humidity for 48 h, corresponding to 1 parasite replication cycle. Parasitemia was measured using SYBR Green 1X (Sigma Aldrich, St-Quentin-Fallavier, France) after facilitating cell membrane disruption with lysis buffer [20 mM Tris (Sigma Aldrich, St-Quentin-Fallavier, France), 5 mM EDTA (Sigma Aldrich, St-Quentin-Fallavier, France), 0.008% (w/v) saponin (Sigma Aldrich, St-Quentin-Fallavier, France) and 0.08% (v/v) Triton X-100]. The fluorescence absorbance was measured using the microplate SAFAS XENIUS reader (SAFAS Monaco, Monaco) with an excitation wavelength of 480 nm and an emission at 520 nm. Data analysis involved calculating the percentage of inhibition based on OD reading using the formula: [100 - (OD test plasma parasitemia/OD non-immune plasma parasitemia × 100)]. In order to reduce inter-plate variation, normalization factors were applied to the percentage of inhibition using the formula [(ODm PC) – (ODm NC)] / [(OD PC) – (OD NC)] where ODm represents the mean reactivity of PC and NC for all plates, and OD PC and OD NC are the mean reactivities of the control plasma samples for a specific plate.

### Statistical analyses

A quantitative comparison of the *P.f* and *T.g* antibody responses was made with a one-way analysis of variance (ANOVA) in multiple comparisons using a Tukey correction. A Pearson correlation was carried out in order to evaluate the linear correlation between *P.f* and *T.g* antibody responses in the case of the malaria-exposed population groups (PDC and COA). Graphics were generated using the software GraphPad Prism version 10.

Linear or logistic regressions were conducted in univariate analyses to identify significant predictors (with *p* ≤ 0.20) to be included in subsequent step-by step multivariate analyses. The statistical analyses comprised various regression models to identify, in each population group, independent variables associated with either functional antibody response (GIA), positive toxoplasmosis serology or positive malaria serology. An interaction between *P.f* and *T.g* antibody response variables was added to evaluate their combined effects in the final multivariate model. Analyses were performed using Stata version 13 (StatCorp LP, TX, USA). Statistical significance was set at *p* ≤ 0.05.

### Ethics approval

The TOXODIAG study “*New diagnostic approach for congenital toxoplasmosis*” received the ethical clearance of the Comité de Protection des Personnes (CPP) Sud-Méditerranée II from France on October 3, 2017, under the ID RCB 2017-A02208-45. The CoaLa study “*Caractérisation de la production d’immunOglobulines spécifiques d’antigènes pArasitaires par les Lymphocytes B néonAtaux dans le cadre d’infections congénitales*” was authorized by the Comité National d’Ethique pour la Recherche en Santé́ (CNERS) from Benin, with the n°40 assigned on September 28, 2017. Similarly, the PlasDCty study “*Elucider et contourner la tolérance immunitaire au paludisme*” was authorized by the CNERS under the n°4 allocated on March 23, 2021. For each study, enrolled women signed an informed written consent before their inclusion and approved the use of the collected data. They were explicitly given the choice to withdraw from each study at any stage. Each study followed the WMA Declaration of Helsinki, adhering to ethical principles for medical research involving human subjects. All the methods were performed in accordance with the approved guidelines and regulations.

## Electronic supplementary material

Below is the link to the electronic supplementary material.

Supplementary Material 1

## Data Availability

The data that support the findings of this study are available from the corresponding authors (M. D and F.M.-N.), upon reasonable request regarding information that could compromise the privacy of research participants.
