# Supplementary material for: Functionality of Toxoplasma gondii antibodies in a population of Beninese pregnant women exposed to malaria
Source: Sci Rep. 2025 Mar 18;15:9303. doi: 10.1038/s41598-025-91803-5 (PMC11920409; doi:10.1038/s41598-025-91803-5)
Supplement: Supplementary file 1 — Supplementary Material 1 [file 41598_2025_91803_MOESM1_ESM.docx]

| *Pf*AMA1 | **N** | **Y** | **M** | **G** | **N** | **P** | **W** | **T** | **E** | **-** | **-** | **-** | **-** | **-** | **-** | **Y** | **M** | **A** | **K** | **Y** | **D** | **I** | **E** | **E** | **V** | **H** | **G** | **S** | **G** | **I** | **R** | **V** | **D** | **L** | **G** | **E** | **D** | **A** | **E** | **V** | **A** | **G** | **T** | **Q** | **Y** | **R** | **L** | **P** | **S** | **G** | [147] |
| --- | --- | --- | --- | --- | --- | --- | --- | --- | --- | --- | --- | --- | --- | --- | --- | --- | --- | --- | --- | --- | --- | --- | --- | --- | --- | --- | --- | --- | --- | --- | --- | --- | --- | --- | --- | --- | --- | --- | --- | --- | --- | --- | --- | --- | --- | --- | --- | --- | --- | --- | --- |
| *Tg*AMA1 | **-** | **-** | **-** | **G** | **N** | **P** | **F** | **Q** | **A** | **N** | **V** | **E** | **M** | **K** | **T** | **F** | **M** | **E** | **R** | **F** | **N** | **L** | **T** | **H** | **H** | **H** | **Q** | **S** | **G** | **I** | **Y** | **V** | **D** | **L** | **G** | **Q** | **D** | **K** | **E** | **V** | **D** | **G** | **T** | **L** | **Y** | **R** | **E** | **P** | **A** | **G** | [115] |
|  |  |  |  |  |  |  |  |  |  |  |  |  |  |  |  |  |  |  |  |  |  |  |  |  |  |  |  |  |  |  |  |  |  |  |  |  |  |  |  |  |  |  |  |  |  |  |  |  |  |  |  |
| *Pf*AMA1 | **K** | **C** | **P** | **V** | **F** | **G** | **K** | **G** | **I** | **I** | **I** | **E** | **N** | **S** | **N** | **-** | **-** | **-** | **-** | **-** | **T** | **T** | **F** | **L** | **T** | **P** | **V** | **A** | **T** | **G** | **Q** | **Y** | **L** | **K** | **D** | **-** | **-** | **-** | **-** | **-** | **-** | **G** | **G** | **F** | **A** | **F** | **-** | **-** | **-** | **P** | [184] |
| *Tg*AMA1 | **L** | **C** | **P** | **I** | **W** | **G** | **K** | **H** | **I** | **E** | **L** | **Q** | **Q** | **P** | **D** | **R** | **P** | **P** | **Y** | **R** | **N** | **N** | **F** | **L** | **E** | **D** | **V** | **P** | **T** | **E** | **K** | **E** | **Y** | **K** | **Q** | **S** | **G** | **N** | **P** | **L** | **P** | **G** | **G** | **F** | **N** | **L** | **N** | **F** | **V** | **T** | [165] |
|  |  |  |  |  |  |  |  |  |  |  |  |  |  |  |  |  |  |  |  |  |  |  |  |  |  |  |  |  |  |  |  |  |  |  |  |  |  |  |  |  |  |  |  |  |  |  |  |  |  |  |  |
| *Pf*AMA1 | **P** | **T** | **E** | **P** | **L** | **M** | **S** | **P** | **M** | **T** | **L** | **D** | **E** | **M** | **R** | **H** | **F** | **Y** | **K** | **D** | **N** | **K** | **Y** | **V** | **K** | **N** | **L** | **D** | **E** | **L** | **T** | **L** | **C** | **S** | **R** | **H** | **A** | **G** | **N** | **M** | **I** | **P** | **-** | **-** | **D** | **N** | **D** | **K** | **N** | **S** | [223] |
| *Tg*AMA1 | **P** | **S** | **G** | **Q** | **R** | **I** | **S** | **P** | **F** | **P** | **M** | **E** | **L** | **L** | **-** | **-** | **-** | **-** | **-** | **E** | **N** | **S** | **N** | **I** | **K** | **A** | **S** | **T** | **D** | **L** | **G** | **R** | **C** | **A** | **E** | **F** | **A** | **F** | **K** | **T** | **V** | **A** | **M** | **D** | **K** | **N** | **N** | **K** | **A** | **T** | [211] |
|  |  |  |  |  |  |  |  |  |  |  |  |  |  |  |  |  |  |  |  |  |  |  |  |  |  |  |  |  |  |  |  |  |  |  |  |  |  |  |  |  |  |  |  |  |  |  |  |  |  |  |  |
| *Pf*AMA1 | **N** | **Y** | **K** | **Y** | **P** | **A** | **V** | **Y** | **D** | **D** | **K** | **D** | **K** | **K** | **C** | **H** | **I** | **L** | **Y** | **I** | **A** | **A** | **Q** | **E** | **N** | **N** | **G** | **P** | **R** | **Y** | **C** | **M** | **-** | **-** | **-** | **-** | **-** | **-** | **-** | **-** | **-** | **-** | **F** | **C** | **F** | **R** | **P** | **A** | **K** | **D** | [281] |
| *Tg*AMA1 | **K** | **Y** | **R** | **Y** | **P** | **F** | **V** | **Y** | **D** | **S** | **K** | **K** | **R** | **L** | **C** | **H** | **I** | **L** | **Y** | **V** | **S** | **M** | **Q** | **L** | **M** | **E** | **G** | **K** | **K** | **Y** | **C** | **S** | **V** | **K** | **G** | **E** | **P** | **P** | **D** | **L** | **T** | **W** | **Y** | **C** | **F** | **K** | **P** | **R** | **K** | **S** | [261] |
|  |  |  |  |  |  |  |  |  |  |  |  |  |  |  |  |  |  |  |  |  |  |  |  |  |  |  |  |  |  |  |  |  |  |  |  |  |  |  |  |  |  |  |  |  |  |  |  |  |  |  |  |
| *Pf*AMA1 | **I** | **S** | **F** | **Q** | **N** | **Y** | **-** | **-** | **-** | **-** | **-** | **T** | **Y** | **L** | **S** | **K** | **N** | **V** | **V** | **D** | **N** | **W** | **E** | **K** | **V** | **C** | **P** | **R** | **K** | **N** | **L** | **Q** | **N** | **A** | **K** | **F** | **G** | **L** | **W** | **V** | **D** | **G** | **N** | **C** | **E** | **D** | **I** | **P** | **H** | **V** | [325] |
| *Tg*AMA1 | **V** | **T** | **E** | **N** | **H** | **H** | **L** | **I** | **Y** | **G** | **S** | **A** | **Y** | **V** | **G** | **E** | **N** | **-** | **P** | **D** | **A** | **F** | **I** | **S** | **K** | **C** | **P** | **N** | **Q** | **A** | **L** | **R** | **G** | **Y** | **R** | **F** | **G** | **V** | **W** | **K** | **K** | **G** | **R** | **C** | **L** | **D** | **Y** | **T** | **E** | **L** | [310] |
|  |  |  |  |  |  |  |  |  |  |  |  |  |  |  |  |  |  |  |  |  |  |  |  |  |  |  |  |  |  |  |  |  |  |  |  |  |  |  |  |  |  |  |  |  |  |  |  |  |  |  |  |
| *Pf*AMA1 | **N** | **E** | **F** | **P** | **A** | **I** | **D** | **L** | **F** | **E** | **-** | **-** | **-** | **-** | **C** | **N** | **K** | **L** | **V** | **F** | **E** | **L** | **S** | **-** | **-** | **A** | **S** | **D** | **Q** | **P** | **K** | **Q** | **Y** | **E** | **Q** | **H** | **L** | **T** | **D** | **Y** | **E** | **K** | **I** | **K** | **E** | **G** | **F** | **K** | **N** | **K** | [370] |
| *Tg*AMA1 | **T** | **D** | **-** | **T** | **V** | **I** | **E** | **R** | **V** | **E** | **S** | **K** | **A** | **Q** | **C** | **W** | **V** | **K** | **T** | **F** | **E** | **N** | **D** | **G** | **V** | **A** | **S** | **D** | **Q** | **P** | **H** | **T** | **Y** | **P** | **-** | **-** | **L** | **T** | **S** | **Q** | **A** | **S** | **W** | **N** | **D** | **W** | **W** | **P** | **-** | **-** | [355] |
|  |  |  |  |  |  |  |  |  |  |  |  |  |  |  |  |  |  |  |  |  |  |  |  |  |  |  |  |  |  |  |  |  |  |  |  |  |  |  |  |  |  |  |  |  |  |  |  |  |  |  |  |
| *Pf*AMA1 | **N** | **A** | **S** | **M** | **I** | **K** | **S** | **A** | **F** | **L** | **P** | **T** | **A** | **D** | **R** | **Y** | **K** | **S** | **H** | **G** | **K** | **G** | **Y** | **N** | **W** | **G** | **N** | **Y** | **N** | **T** | **E** | **T** | **Q** | **-** | **-** | **-** | **K** | **C** | **E** | **I** | **F** | **N** | **V** | **K** | **P** | **T** | **C** | **L** | **I** | **N** | [421] |
| *Tg*AMA1 | **-** | **-** | **-** | **-** | **-** | **-** | **-** | **-** | **-** | **L** | **H** | **Q** | **S** | **D** | **Q** | **P** | **H** | **S** | **G** | **G** | **V** | **G** | **R** | **N** | **Y** | **G** | **F** | **Y** | **Y** | **V** | **D** | **T** | **T** | **G** | **E** | **G** | **K** | **C** | **A** | **L** | **S** | **D** | **Q** | **V** | **P** | **D** | **C** | **L** | **V** | **S** | [395] |
|  |  |  |  |  |  |  |  |  |  |  |  |  |  |  |  |  |  |  |  |  |  |  |  |  |  |  |  |  |  |  |  |  |  |  |  |  |  |  |  |  |  |  |  |  |  |  |  |  |  |  |  |
| *Pf*AMA1 | **N** | **S** | **-** | **-** | **-** | **S** | **Y** | **I** | **A** | **T** | **T** | **A** | **L** | **S** | **-** | **-** | **-** | **-** | **-** | **-** | **-** | **-** | **-** | **-** | **-** | **-** | **-** | **-** | **-** | **-** | **-** | **-** | **-** | **-** | **-** | **-** | **-** | **-** | **-** | **-** | **H** | **P** | **-** | **-** | **-** | **-** | **-** | **I** | **E** | **V** | [437] |
| *Tg*AMA1 | **D** | **S** | **A** | **A** | **V** | **S** | **Y** | **T** | **A** | **A** | **G** | **S** | **L** | **S** | **E** | **E** | **T** | **P** | **N** | **F** | **I** | **I** | **P** | **S** | **N** | **T** | **P** | **P** | **T** | **P** | **E** | **T** | **A** | **L** | **Q** | **C** | **T** | **A** | **D** | **K** | **F** | **P** | **D** | **S** | **F** | **G** | **A** | **C** | **D** | **V** | [449] |
|  |  |  |  |  |  |  |  |  |  |  |  |  |  |  |  |  |  |  |  |  |  |  |  |  |  |  |  |  |  |  |  |  |  |  |  |  |  |  |  |  |  |  |  |  |  |  |  |  |  |  |  |
| *Pf*AMA1 | **E** | **-** | **-** | **-** | **-** | **-** | **-** | **-** | **-** | **-** | **-** | **-** | **-** | **-** | **-** | **-** | **-** | **-** | **-** | **-** | **-** | **-** | **-** | **-** | **-** | **-** | **-** | **-** | **-** | **-** | [438] | | |  |  |  |  |  |  |  |  |  |  |  |  |  |  |  |  |  |  |
| *Tg*AMA1 | **Q** | **A** | **C** | **K** | **R** | **Q** | **K** | **T** | **S** | **C** | **V** | **G** | **G** | **Q** | **I** | **Q** | **S** | **T** | **S** | **V** | **D** | **C** | **T** | **A** | **D** | **E** | **Q** | **N** | **E** | **C** | [479] | | |  |  |  |  |  |  |  |  |  |  |  |  |  |  |  |  |  |  |

**SUPPLEMENTARY DATA**

**Supp. Table 1: Linear alignment of the *Pf*AMA1 and *Tg*AMA1 antigens with their respective predicted B epitopes.**

The amino-acid linear sequence of *Pf*AMA1 PDB ref 4R19 (104 to 438) is aligned with *Tg*AMA1 PDB ref 2X2Z (69 to 479). The PDB alignment algorithm displays 32% of identity with conserved sites, as shown in yellow. Regions with predicted discontinuous epitopes are indicated in red for *Pf*AMA1 and in orange for *Tg*AMA1.

**Supp. Table 2: Univariate linear regression analysis of the combined malaria and toxoplasmosis serological status in relation to specific IgG responses against *P.f* and *T.g* antigens**

|  | IgG anti-*Pf*AMA1 | |  | IgG anti-*Tg*AMA1 | |  | IgG anti-*Tg*SAG1 IgG | |  | IgG anti-*Tg*GRA7 | |  | IgG anti-*Pf*s48/45 ^d^ | |  |
| --- | --- | --- | --- | --- | --- | --- | --- | --- | --- | --- | --- | --- | --- | --- | --- |
| *Variables (n=148)* ^a^ | *coef^b^* | *95% CI* | *pval* | *coef^b^* | *95% CI* | *pval* | *coef^b^* | *95% CI* | *pval* | *coef^b^* | *95% CI* | *pval* | *coef^b^* | *95% CI* | pval |
| Serological Status ^c^ |  |  |  |  |  |  |  |  |  |  |  |  |  |  |  |
| Malaria (+) and toxoplasmosis (-) | 1.23 | 0.11 2.34 | **0.031** | 0.07 | -0.31 0.45 | 0.706 | -0.44 | -0.75 -0.13 | **0.005** | 0.22 | -0.17 0.60 | 0.269 | 1.22 | 0.74 1.70 | **<0,001** |
| Malaria (+) and toxoplasmosis (+) | 2.14 | 1.04 3.25 | **<0,001** | 1.98 | 1.60 2.36 | **<0,001** | 2.53 | 2.22 2.83 | **<0,001** | 1.41 | 1.03 1.80 | **<0,001** | 1.01 | 0.54 1.49 | **<0,001** |
| Malaria (-) and toxoplasmosis (+) | 0.34 | -1.07 1.75 | 0.631 | 1.42 | 0 .93 1.90 | **<0,001** | 2.01 | 1.62 2.40 | **<0,001** | 1.14 | 0.66 1.63 | **<0,001** | 0.1 | -0.50 0.70 | 0.743 |

^a^: n=148 including PDC (n=29), COA (n=95) and TXD (n=26 minus 2 individuals with missing data); ^b^: coef = a positive coefficient indicates an increase of both independent and dependent variables. A negative coefficient indicates an increase of the independent while the dependent variable tends to decrease; ^C^: the combined status “malaria and toxoplasmosis negative serology” is the reference; ^d^: n = 119, the evaluation of antibody response against *Pf*s48/45 was only performed in COA (n=95) and TXD groups (n=26 minus 2 individuals with missing data).

**Supp.Table 3 : Individual growth inhibiton values for the three population groups**

**Supp. Figure 1: Structural alignment of *Pfs*48/45 and *Tg*SAG1.**The domain DIII of *Pf*s48/45 (7ZXG_D) is aligned with the domain DI of *Tg*SAG1 structure (1KZQ_A). RMSD=3.0, TM score=0.38, identity=19%.

**Supp. Figure 2: Quantitative analysis of the IgG responses directed to *T.g* and *P.f* antigens according to the toxoplasmosis serology from the pregnant women under study.** Toxoplasmosis seropositive group: PDC n=16; COA n=45; TXD n=11. Toxoplasmosis seronegative group: PDC n=13; COA n=50; TXD n=15. The cut-off for every specific antibody response is represent by a dotted line.


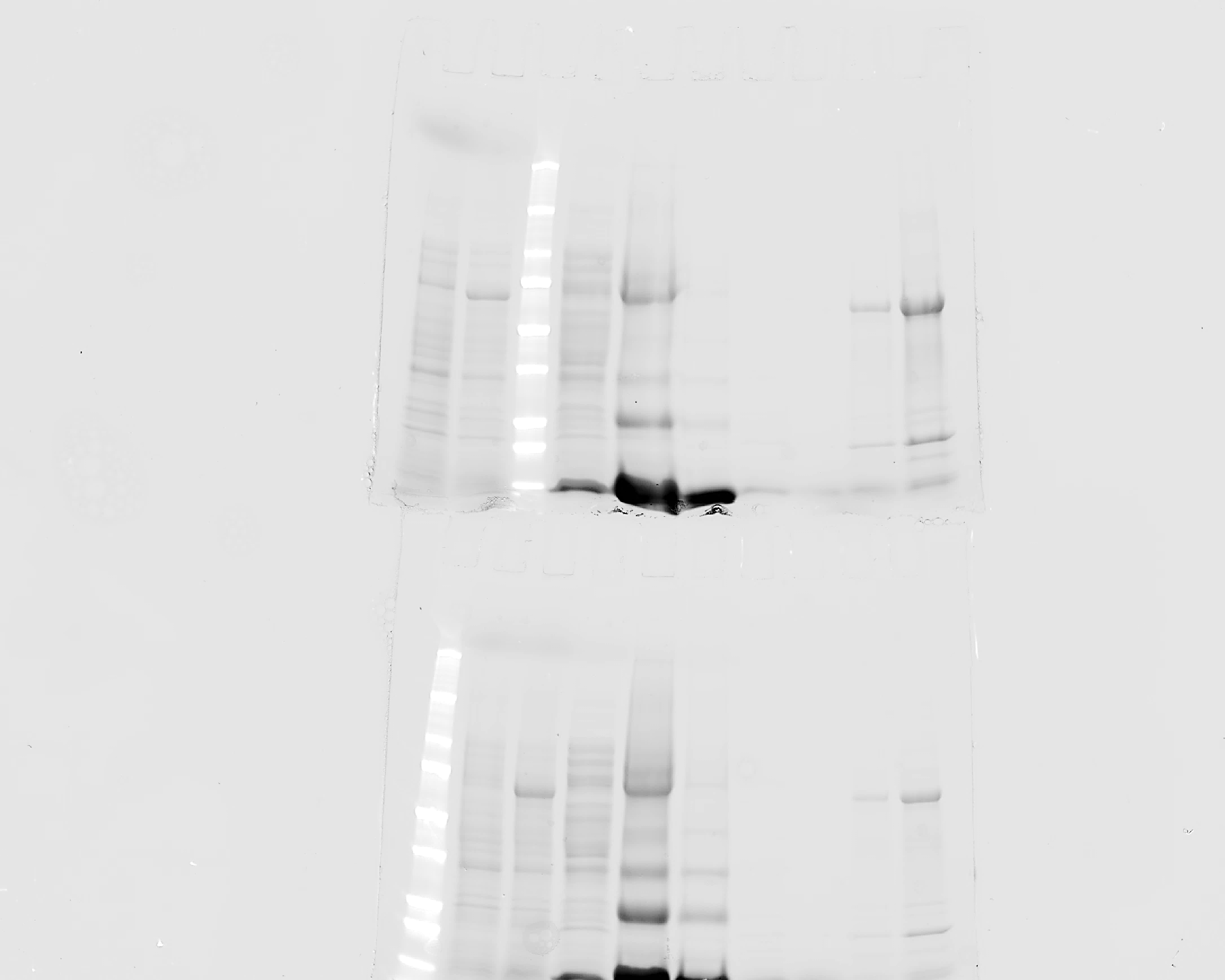


**1**

**2**

**3**

**4**

**5**

**6**

**7**

**8**

**9**

**10**

**W3**

**W2**

**W1**

**S**

**P**

**I**

**U**

**MW**

**C**

**E**

**37**

**100**

***Pf*AMA1**

**50**

**75**

**150**

**250**

**10**

**25**

**20**

**Supp. Figure 3: SDS-PAGE gel electrophoresis analysis of the recombinant antigen *Pf*AMA1**. An SDS PAGE gel (Bio Rad) was performed using the technology stain free (ChemiDoc Imaging System, Bio Rad) to visualise the produced protein *Pf*AMA1 (~75kDa) during the process of induction and purification. MW= Molecular Weight; U= Uninduced; I=Induced; S= Supernatant; P= Pellet; W1, W2, W3= Washing steps; E= Eluted protein; C= Concentrated protein.


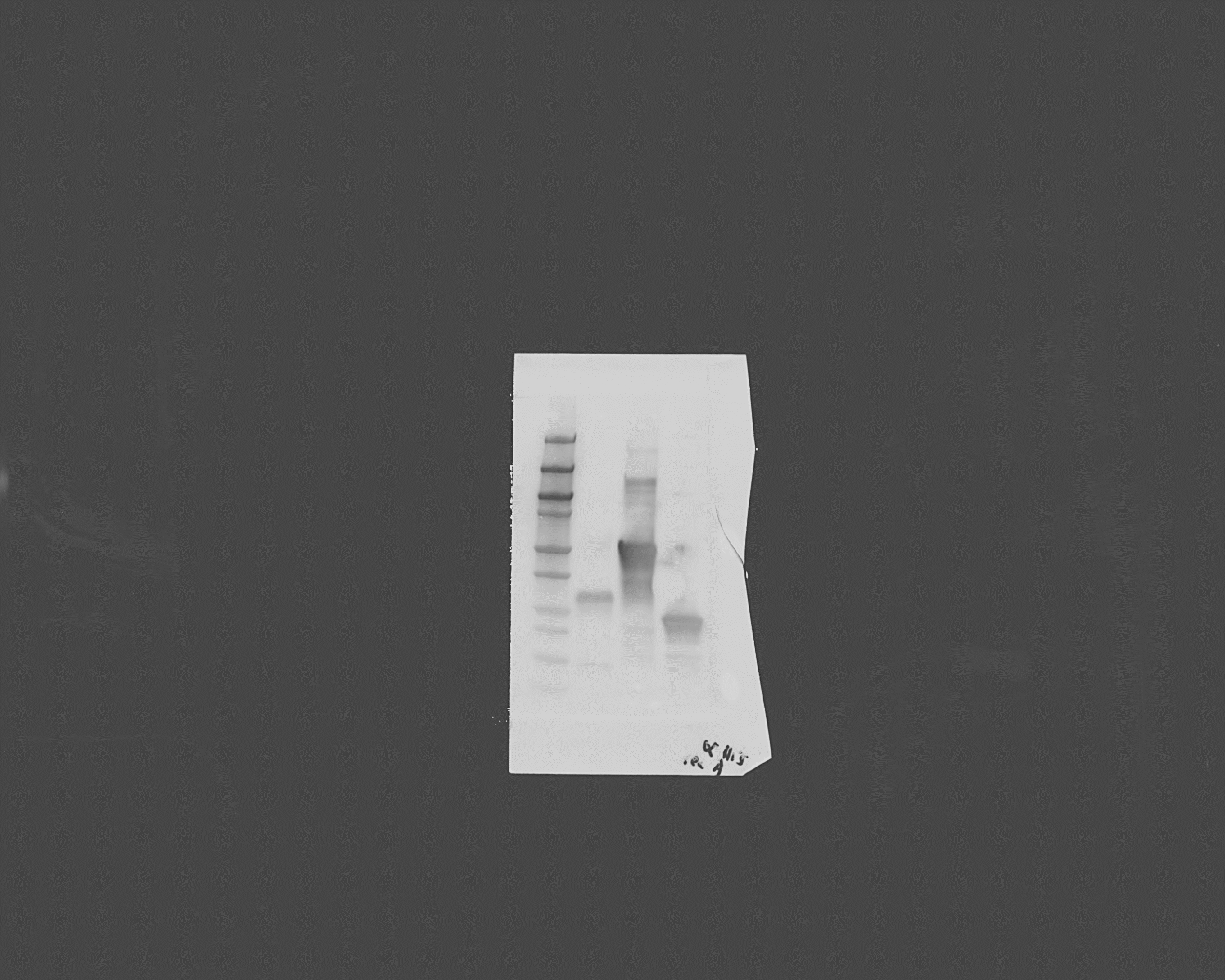

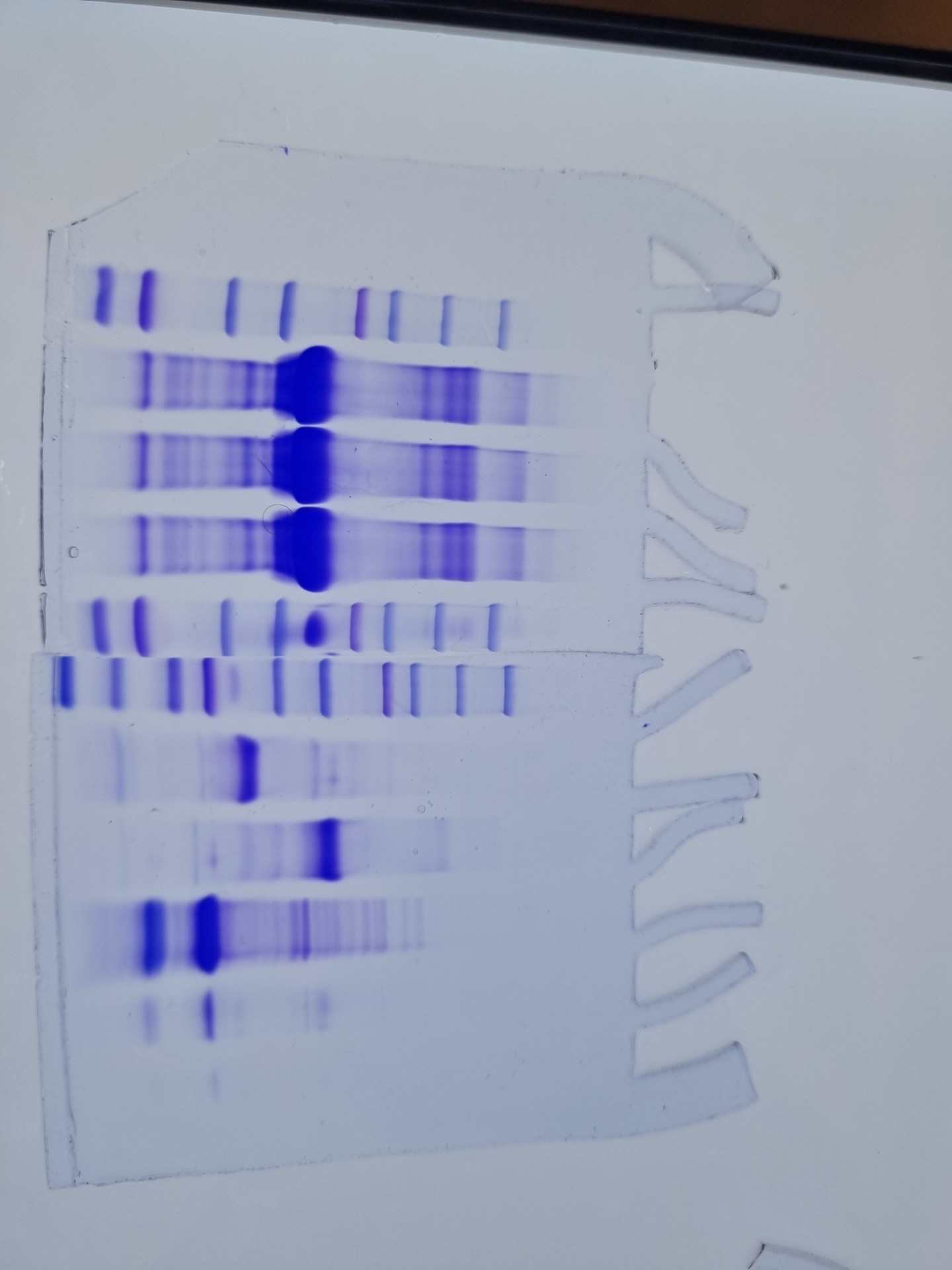


***Tg*SAG1**

**B**

**10**

***Tg*GRA7**

***Tg*AMA1**

**MW**

**15**

**20**

**25**

**37**

**50**

**75**

**100**

**150**

**250**

***Tg*GRA7***

**A**

**10**

**15**

**20**

**25**

**37**

**50**

**75**

**250**

**100**

**150**

**MW**

***Tg*SAG1**

***Tg*AMA1**

***Tg*GRA7**

**Supp. Figure 4: Results of the *T. gondii* purified recombinant antigens.** Coomassie blue gel **(A)** and anti-His Western blot **(B)** of the purified recombinant antigens. A half nitrocellulose membrane onto which recombinant proteins were transferred was incubated with an anti-His HRP secondary antibody (Penta·His Antibody, QIAGEN) at 1:5000 in PBS1X-BSA 1%. *Tg*SAG1 (31kDa), *Tg*AMA1 (50kDa) and *Tg*GRA7 (25 kDA); the 18kDa band corresponds to *Tg*GRA7* degradation products. MW = Molecular Weight (Precision Plus Protein™ Dual Color Standards, BioRad).
